# LearningRx Cognitive Training Effects in Children Ages 8–14: A Randomized Controlled Trial

**DOI:** 10.1002/acp.3257

**Published:** 2016-08-02

**Authors:** Dick M. Carpenter, Christina Ledbetter, Amy Lawson Moore

**Affiliations:** ^1^ University of ColoradoColorado SpringsUSA; ^2^ Louisiana State UniversityHealth Sciences CenterShreveportUSA; ^3^ Gibson Institute of Cognitive ResearchColorado SpringsUSA

## Abstract

In a randomized controlled study, we examined the effects of a one‐on‐one cognitive training program on memory, visual and auditory processing, processing speed, reasoning, attention, and General Intellectual Ability (GIA) score for students ages 8–14. Participants were randomly assigned to either an experimental group to complete 60 h of cognitive training or to a wait‐list control group. The purpose of the study was to examine changes in multiple cognitive skills after completing cognitive training with ThinkRx, a LearningRx program. Results showed statistically significant differences between groups on all outcome measures except for attention. Implications, limitations, and suggestions for future research are examined. © 2016 The Authors *Applied Cognitive Psychology* Published by John Wiley & Sons Ltd.

The modification of ‘IQ’ has been an elusive quest of many neuroplasticity researchers who have found little transfer from targeted cognitive training interventions to general intelligence (Chein & Morrison, 2010; Dunning, Holmes, & Gathercole, 2013). Although transfer from working memory training to fluid intelligence has been documented in several small studies (Jaeggi, Buschkuehl, Jonides, & Perrig, 2008; Jaušovec & Jaušovec, 2012), skepticism continues to permeate the field (Redick et al., 2013). This bent is understandable given the number of non‐significant findings. Despite the controversy, the modern brain training movement has exploded with an assortment of programs designed to enhance cognitive function. The purpose of the present study was to examine the effects of a one‐on‐one cognitive training program on General Intellectual Ability (GIA) as well as on fluid reasoning, memory, visual and auditory processing, processing speed, and attention—all key cognitive skills that underlie the ability to learn.

Extant research has demonstrated support for the efficacy of cognitive training programs in improving individual cognitive skills (Holmes, Gathercole, & Dunning, 2009; Klingberg et al., 2005; Melby‐Lervag & Hulme, 2013; Sonuga‐Barke et al., 2013; Wegrzyn, Hearrington, Martin, & Randolph, 2012). However, because each training program described in the literature targets different cognitive skills, the results are as diverse and varied as the programs themselves. Given the growing research base on the associations among working memory and intelligence (Cornoldi & Giofre, 2014), and working memory and learning (Alloway & Copello, 2013), it is easy to see why a majority of the cognitive training programs target working memory. Certainly, most of the studies do cite improvements in working memory (Beck, Hanson, & Puffenberger, 2010; Dunning et al., 2013; Gray et al., 2012; Holmes & Gathercole, 2014; Wiest, Wong, Minero, & Pumaccahua, 2014), but pretest to post‐test gains have also been documented in fluid reasoning (Barkl, Porter, & Ginns, 2012; Jaeggi et al., 2008; Mackey, Hill, Stone, & Bunge, 2011), processing speed (Mackey et al., 2011), reading (Loosli, Buschkuehl, Perrig, & Jaeggi, 2012; Shalev, Tsal, & Mevorach, 2007), computational accuracy (Witt, 2011), and attention (Rabiner, Murray, Skinner, & Malone, 2010; Tamm, Epstein, Peugh, Nakonezny, & Hughes, 2013).

Despite the assertion that fluid intelligence and individual cognitive skills can be trained (Sternberg, 2008), the evidence that IQ scores can be modified by a training intervention is scarce. An intriguing gap in the literature is the dearth of cognitive training studies that specifically measure effects on IQ score, especially given the role of IQ scores in predicting reading ability (Naglieri & Ronning, 2000), academic achievement (Freberg, Vandiver, Watkins, & Canivez, 2008), the severity of children's mental health problems (Mathiassen et al., 2012), social mobility (Forrest, Hodgson, Parker, & Pearce, 2011), obesity (Chandola, Deary, Blane, & Batty, 2006), suicidality (Gunnell, Harbord, Singleton, Jenkins, & Lewis, 2009), early mortality (Maenner, Greenberg, & Mailick, 2015), income potential (Murray, 2002), and occupational performance (Hunter, 1986). The assessment of GIA—although standard practice in the formal diagnoses of learning disabilities—can provide valuable information as a response to intervention context as well. Anastasi and Urbina (1997) suggest that intelligence tests should be used to assess strengths and weaknesses in order to plan how to bring people to their maximum level of functioning.

The implicit measurement of general intelligence is hinted at in the studies using tests of fluid reasoning. Barkl et al. (2012) argue that the high correlation between fluid reasoning and general intelligence supports the assumption that interventions targeting fluid reasoning will necessarily target IQ score. While their findings included significant improvements in inductive reasoning following reasoning training, a comprehensive measure of GIA was not included in the study. Hayward, Das, and Janzen (2007) used the full scale score on the Das–Naglieri Cognitive Assessment System (CAS) in their study of the COGENT cognitive training program but did not find significant group differences on the measure. Dunning et al. (2013) included the Wechsler Abbreviated Scales of Intelligence in their measures of working memory training outcomes and found no evidence that training working memory leads to enhancement in non‐verbal intelligence score. This finding that the training did not impact IQ score was consistent with findings from a previous study on the same working memory training program (Holmes et al., 2010). Although Roughan and Hadwin (2011) did note significant group differences in IQ score as measured by Raven's Standard Progressive Matrices in a small study (*n* = 15), Mansur‐Alves and Flores‐Mendoza (2015) did not find significant post‐training differences between groups on the Raven's test in a larger study (*n* = 53). Thus, the lack of corroborating findings presents an unconvincing view that working memory training alone is a tool for increasing IQ score.

Xin, Lai, Li, and Maes (2014) suggest that the mixed results from working memory training studies may be because of the differences in working memory tasks used in the interventions. Harrison, Shipstead, and Engle (2015) propose that the relationship between working memory and fluid intelligence is a function of the matrix tasks used to measure fluid intelligence. Specifically, the ability to maintain solutions from prior items on the Raven's in active memory will enhance performance on the test. Alternatively, perhaps the inconsistency in findings is not associated with variations in tasks, in the ability to recycle solutions, or in working memory training efficacy per se, but in the narrow theoretical foundation on which working memory training programs may be based. With few exceptions, the commercially available programs are based on Baddeley's (1992) model of working memory—the three‐component model showcasing the phonological loop, visuo‐spatial sketchpad, and central executive as the most widely accepted theory of working memory. However, given that development and revision of contemporary IQ tests are guided by the ever‐evolving Cattell–Horn–Carroll (CHC) theory of cognitive abilities (McGrew, 2009), it should follow that interventions grounded in a similar theoretical basis should have a larger impact on IQ score and the multiple cognitive constructs on which a full scale IQ score is collectively determined. The CHC theory is a relatively new model of intelligence that merges the Gf‐Gc theory (fluid intelligence and crystallized intelligence, respectively) espoused by Cattell and Horn (1991) and the tri‐stratum model of intelligence espoused by Carroll (1993). The most recent update of the model (Schneider & McGrew, 2012) places the individual cognitive abilities in four categories: acquired knowledge (crystallized intelligence), domain‐independent general capacities (fluid reasoning and memory), sensory‐motor abilities (visual and auditory processing), and general speed (processing speed, reaction times, and psychomotor speed)—all under the umbrella of GIA. Thus, it would be interesting to investigate if comprehensive cognitive training interventions that target multiple cognitive abilities across these categories have an effect not only on the individual cognitive constructs but also on a *GIA* score (McGrew, Schrank, & Woodcock, 2007).

One such program has been developed to target multiple cognitive abilities. As described in a prior study (Gibson, Carpenter, Moore, & Mitchell, 2015), the ThinkRx cognitive training program targets and remediates seven general cognitive skills and 25 subskills through repeated engagement in game‐like mental tasks delivered one‐on‐one by a cognitive trainer (Table 1). The 60‐h program includes a 230‐page curriculum consisting of 23 different training procedures with more than 1000 total difficulty levels. The program components are sequenced and loaded by difficulty and intensity. Trainers use a metronome, stopwatch, tangrams, shape and number cards, workboards, a trampoline, footbag, and activity sheets to deliver the program to students. The training tasks emphasize visual or auditory processes that require attention and reasoning throughout each 60 to 90‐min training period. Training sessions are focused, demanding, intense, and tightly controlled by the trainer to push students to just above their current cognitive skill levels. Deliberate distractions are built in to the sessions to tax the brain's capacity for sorting and evaluating the importance of incoming information. The use of a metronome increases intensity and ensures there are no ‘mental breaks’ while completing a training task.

**Table 1:** Descriptions of training tasks and skills targeted by each task, and the number of difficulty levels in each task

Task description	Skills targeted	Levels
1. Colored arrows or words are displayed. Participants call out colors, directions, or words	DA, PS, SA, STA, VM, VN, WM	48
2. Columns of numbers are displayed. Participants add, subtract, or multiply a constant number to each number in the column.	PS, MC, DA, LTM, STA, WM	35
3. A more difficult version of #2 using multiple operations and optional trampoline.	PS, SF, MC, DA, LTM, STA, VS, WM	44
4. Participants visually fixate on a pen while simultaneously completing a mental activity	STA, VP, DA, VF, SM	18
5. Participants perform actions on charts of numbers and letters.	PS, DA, MC, WM, SF, SA, SM, STA, VD, VS	44
6. Participants are asked to paraphrase stories and represent concepts with concrete objects.	VN, C, SP, SSP, LR	17
7. Participants listen to or read descriptors and select the object that matches the descriptions.	VN, C, LR, SP, WM	32
8. Trainer and participant toss a hacky sack on metronome beat	AA, DA, MC, PS, SM, WM, SSA, VN	5
9. Participant claps and taps in rhythm to the metronome with distractions	AD, DA, SA, SM, SP, SSA	13
10. Participant touches his thumb to his fingers on beat with mental activities	DA, PS, SM	6
11. Participant studies numbers and their positions on a card and recalls the digits and positions on beat	DA, MC, WM, VP, VS, VN	25
12. Trainer calls off numbers for participant to perform a mathematical operation on n‐back numbers using a timer and metronome	DA, C, PS, SA, SSA, WM, SP	44
13. Participant studies patterns of shapes and reproduces them from memory	WM, LTM, VD, VS, SSA, PS, SP	35
14. Participant identifies three‐card groups sharing shape, color, orientation, and size characteristics	LR, C, WM, PS, SA, SSA, VD	40
15. Participant reasons through brain teaser cards	LR, VN, C, SP, VM	32
16. Trainer and participant visualize and verbally play tic tac toe activities	DA, EP, PS, SP, VP, STM, VN	32
17. Using a golf course map, participant studies the route to the hole and draws the route with his eyes closed.	VP, VN, SM	32
18. Participant studies humorous images representing groups of related people, objects, numbers, and concepts and recalls the items from memory	LTM, VN, AM, C, PS, SSA, WM, VP	34
19. Participant recreates studied images with tangrams	VN, LR, SP, SM, STM, SP, SSA, VM, WM	38
20. Participant visualizes and spells words in the air	VP, VN, WM	6
21. Trainer drills participant on 17 sounds	AA, AD, AP	14
22. Participant segments sounds of words	AA, AD, AP, AS	14
23. Participant blends sounds to make words	AA, AB, AD, AP	14
24. Participate manipulates words by removing sounds	AA, AD, AP	14

Note. *AA = auditory analysis, AB = auditory blending, AD = auditory discrimination, AP = auditory processing, AS = auditory segmenting, AM = associative memory, C = comprehension, DA = divided attention, EP = executive processing, LR = logic and reasoning, MC = math computation, PS = processing speed, SF = saccadic fixation, SA = selective attention, SM = sensory‐motor integration, SP = sequential processing, STM = short term memory, SSP = simultaneous processing, STA = sustained attention, VP = visual processing, VD = visual discrimination, VF = visual fixation, VM = visual manipulation, VN = visualization, VS = visual span, WM = working memory.*

Each ThinkRx training procedure targets various combinations of multiple skills such as working memory, processing speed, visualization, auditory discrimination, reasoning, sensory motor integration, and attention. The program itself is grounded in *The Learning Model* (Gibson, Hanson, & Mitchell, 2007; Gibson et al., 2015; Press, 2012), a pictorial representation of information processing shown in Figure 1. *The Learning Model* is based on the CHC theory espousing a multiple‐construct view of intelligence. (For a complete description of CHC Theory, see McGrew, 2005.) The Learning Model illustrates the role of individual cognitive abilities in cognitive skill efficiency and its direct influence on the ability to store and retrieve accumulated knowledge. For example, information is acquired through the senses and must then be recognized and analyzed by a fluid or active processing system that includes working memory, processing speed, and attention. This is the executive control system that determines which information is unimportant, easily handled, or requires more complex processing. If the information is novel or complex, higher order processes such as reasoning, auditory processing, and visual processing must occur in order to complete the task. With practice, higher order processing can be bypassed, which helps decrease the time between sensory input and output.

**Figure 1:**
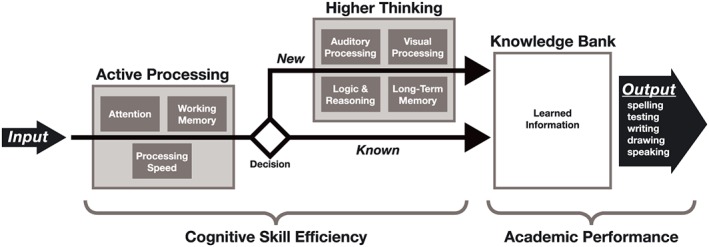
Pictorial representation of The Learning Model

The one‐on‐one delivery method of the ThinkRx program is supported by Feuerstein's theory of structural cognitive modifiability, which posits that cognition is not static but malleable as a result of mediated experiences with the world (Feuerstein, Feuerstein, & Falik, 2010). This mediation represents the role of the adult in the students' ability to make sense of stimuli in the environment. It is not the impartation of knowledge upon a student but, instead, the purposeful coaching of a student's interaction with a stimulus to bring about understanding and build cognitive capacity for learning. Research on Feuerstein's Instrumental Enrichment Basic (IE) cognitive training program suggests that fluid intelligence can be modified through these interactions (Kozulin et al., 2010). As is Feuerstein's program, the trainer‐delivered ThinkRx program is distinct from the computer‐based cognitive training programs that are ubiquitous in the extant literature.

Although statistically significant cognitive skill gains have been noted in four doctoral research studies on the ThinkRx program (Jedlicka, 2012; Luckey, 2009; Moore, 2015; Pfister, 2013), published research on the efficacy of ThinkRx has only recently begun to proliferate. Building on the results from a quasi‐experimental study on the ThinkRx program, which used propensity‐matched controls (Gibson et al., 2015), it was important to conduct a randomized, controlled trial if results of the study were to make a meaningful contribution to the existing literature on cognitive training for remediating deficits in multiple cognitive skills.

## Method

To examine the effects of a one‐on‐one cognitive training program on children's cognitive skills, we conducted a randomized, pretest–posttest control group study using the ThinkRx cognitive training program delivered by cognitive trainers in two training locations. This study was guided by the following question: Is there a statistically significant difference in GIA score, Associative Memory, Visual Processing, Auditory Processing, Logic and Reasoning, Processing Speed, Working Memory, Long Term Memory, and Attention between those who complete ThinkRx cognitive training and those who do not?

### Participants

The sample for the study (*n* = 39) was recruited from the population of students ages 8–14 in a database of families who had requested information about LearningRx cognitive training in Colorado Springs in the three years prior to the study. A recruitment email was sent to all families in the database (*n* = 2241). Eligibility was limited to participants between the ages of 8 and 14 who lived within commuting distance of Colorado Springs and who scored at screening between 70 and 130 on the GIA composite of the Woodcock Johnson III—Tests of Cognitive Abilities. Of the 43 volunteers, 39 students met the criteria for participation. Using blocked sampling with siblings and individuals, participants were randomly assigned to one of two groups: an experimental group that completed 60 h of one‐on‐one cognitive training versus a waitlist control group. Blocking by sibling or individual status was chosen to reduce the risk of attrition and contamination if siblings were assigned to different groups. The experimental group (*n* = 20) included 11 females and nine males, with a mean age of 11.3. In the experimental group, parent‐reported diagnoses included ADHD (*n* = 6), dyslexia (*n* = 3), LD (*n* = 2), speech delay (*n* = 2), and TBI (*n* = 1). The control group (*n* = 19) included seven females and 12 males, with a mean age of 11.1. In the control group, parent‐reported diagnoses included ADHD (*n* = 7), dyslexia (*n* = 3), learning disability (*n* = 1), and speech delay (*n* = 2). Diagnosis was not an exclusion criteria for several reasons. First, prior observational data from LearningRx reveals similar results across diagnostic categories. Next, randomization washes out the influence of diagnosis on training results. Finally, it would be impossible to tease apart differences based on diagnosis in a small sample without losing statistical power. A check of the random assignment indicated the groups were balanced, with no significant differences between groups based on personal characteristics (age: *t* = −.407, *p* = .686; gender: *χ^2^* = 1.29, *p* = .26; race/ethnicity: *χ^2^* = 3.42, *p* = .06; ADD/ADHD: *χ^2^* = .21, *p* = .65; autistic: *χ^2^* = .98, *p* = .32; dyslexia: *χ^2^* = .01, *p* = .95; gifted: *χ^2^* = .42, *p* = .52; LD: *χ^2^* = .31, *p* = .58; none: *χ^2^* = .74, *p* = .39; physical: *χ^2^* = .00, *p* = .97; speech: *χ^2^* = .00, *p* = .96; TBI: *χ^2^* = .96, *p* = .32).

### Training tasks

**Figure 2:**
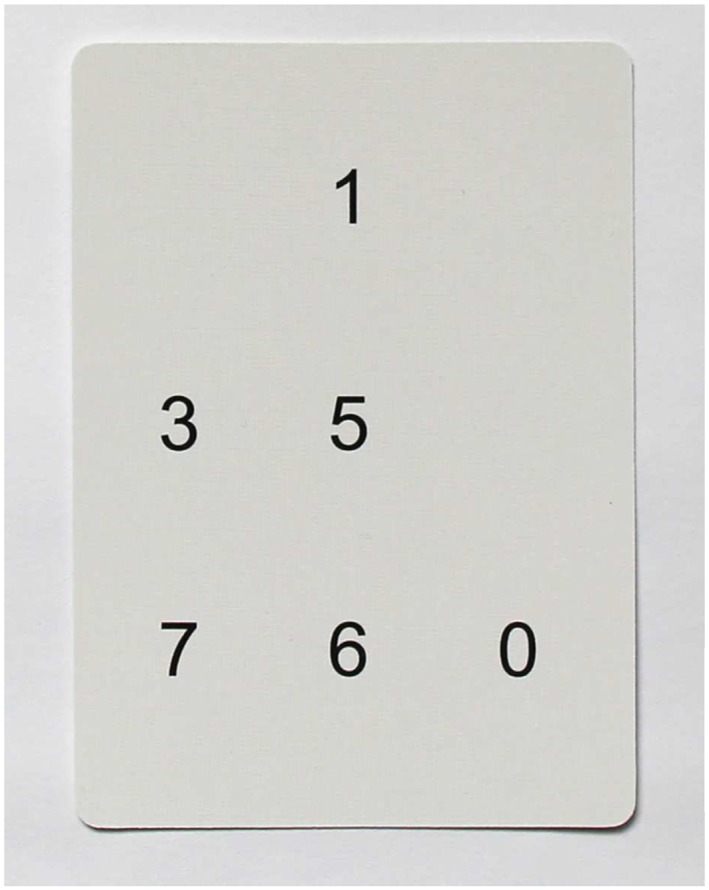
Example of a memory training procedure. Participants study a card for 3 s and then recall the numbers in the correct positions on the grid. Oral responses must be given in beat with the metronome. In this example, the response would be ‘Blank‐1‐blank‐3‐5‐blank‐7‐6‐0’

The ThinkRx cognitive training program includes 23 training tasks. Each task targets a primary cognitive ability and multiple cognitive skills. For example, the primary objective of training task #11 (Figure 2) is to develop working memory but visual span, visualization, and concentration are also developed through this procedure. Descriptions of each training task are presented in Table 1. Trainers tracked participants' progress through each level using a dynamic assessment system. As participants mastered each level of task difficulty, the trainers documented the date and time in individual student workbooks. Trainers provided constant feedback and awarded points for mastery and effort. Participants were able to save and later exchange their points for small prizes or gift cards.

### Testing tasks

#### Associative memory test

The Visual–Auditory Learning subtest of the Woodcock Johnson III—Tests of Cognitive Abilities was administered to measure associative and semantic memory. The test requires encoding and retrieval of auditory and visual associations. The test administrator teaches the participant a rebus, or a set of pictures that each represents a word. Then, the participant must recall the association between the pictures and the words by reading them as a sentence aloud. For ages 5–19, this test has a median reliability of .81 (Mather & Woodcock, 2001).

#### Visual processing test

The Spatial Relations subtest of the Woodcock Johnson III—Tests of Cognitive Abilities measures visual processing skills by asking the student to match individual puzzle pieces to a completed shape. For ages 5–19, this test has a median reliability of .86 (Mather & Woodcock, 2001).

#### Auditory processing test

The Sound Blending subtest of the Woodcock Johnson III—Tests of Cognitive Abilities measures the ability to synthesize phonemes. The test administrator presents a series of phonemes (language sounds) and the student must blend them together to form a word. For ages 5–19, this test has a median reliability of .86 (Mather & Woodcock, 2001).

#### Logic and reasoning test

The Concept Formation subtest of the Woodcock Johnson III—Tests of Cognitive Abilities measures fluid reasoning by requiring the student to use inductive logic and apply rules to sets of shapes that share similarities and differences. The student must indicate the rule that differentiates one set of shapes from the others. For ages 5–19, this test has a median reliability of .94 (Mather & Woodcock, 2001).

#### Working memory test

The Numbers Reversed subtest of the Woodcock Johnson III—Tests of Cognitive Abilities measures working memory by asking the student to remember a span of numbers and repeat them in reverse order from how they were presented. For ages 5–19, this test has a median reliability of .86 (Mather & Woodcock, 2001).

#### Processing speed test

The Visual Matching subtest of the Woodcock Johnson III—Tests of Cognitive Abilities measures perceptual processing speed by asking the student to discriminate visual symbols. In three minutes, the student identifies and circles pairs of matching numbers in each row of six number combinations ranging from single digit to three‐digit numbers. For ages 5–19, this test has a median reliability of .89 (Mather & Woodcock, 2001).

#### Long‐term memory test

The Visual–Auditory Learning‐Delayed subtest of the Woodcock Johnson III—Tests of Cognitive Abilities repeats the verbal–visual associations learned during the Visual–Auditory Learning subtest administered earlier in the testing session. The test requires the student to read the rebus passages again as a measure of long‐term retention. For ages 5–19, this test has a median reliability of .92 (Mather & Woodcock, 2001).

#### Attention test

The Flanker Inhibitory Control and Attention Test from the NIH Toolbox Cognition Battery measures attention and inhibitory control. The computer‐based test requires the student to focus on and identify the direction of an arrow while other arrows are flanking it. For this 3‐min test, scoring is based on a combination of accuracy and reaction time. For ages 8–15, the test has a convergent validity with D‐KEFS Inhibition Test of .34 (Zelazo et al., 2013).

#### GIA score

GIA score is a cluster score on the Woodcock Johnson III—Tests of Cognitive Abilities (Woodcock, McGrew, & Mather, 2001). The score is a weighted composite based on age of seven cognitive skills tests that measure verbal comprehension (20%), associative memory (17%), visual processing (9%), phonemic awareness (12%), fluid reasoning (19%), processing speed (19%), and working memory (13%). Attention and long‐term memory are not included in the GIA score.

### Procedures

After obtaining parental consent, participants were pre‐tested in quiet testing rooms. Under the supervision of a doctoral‐level educational psychologist, master's‐level test administrators assisted with delivering the Woodcock Johnson III—Tests of Cognitive Abilities (1–7, 10) and were blind to the experimental condition. The Flanker Test from the NIH Toolbox—Cognition Battery was administered by trained research assistants. The mean interval from pretest to post‐test was 14.4 weeks for the experimental group and 14.5 weeks for the control group. For the experimental group, participants attended three or four 90‐min training sessions per week during the 15‐week study period for a total of 40 sessions. Training sessions were held at two locations: a cognitive training center and a cognitive science research facility with training rooms similar to those at the training center. LearningRx certified cognitive trainers who were not part of the research team delivered the ThinkRx program during the scheduled sessions. On‐site LearningRx master trainers monitored day‐to‐day program fidelity. The remaining phases of the study including design and data analysis were not performed by LearningRx. One hundred percent of the students in the experimental group completed the required 60‐h protocol and attended all 40 training sessions. The control group participants waited to begin their intervention until the experimental group had completed their 60 h of training. Post‐testing was completed within two weeks of the experimental group's program completion.

### Statistical analysis

Data were analyzed using multivariate analysis of variance (MANOVA), with the dependent variables being the difference scores between the pre and post tests for each measure. In other words, the study used a difference‐in‐difference analysis for all measures. Given the number of pairwise comparisons (i.e., nine, one for each measure), a Bonferroni correction was applied to the multiple comparisons. Effect sizes were also calculated for all measures using Cohen's *d*. To address the potential for Lord's Paradox (Wainer, 1991), we conducted an alternate series of individual analyses of covariance (ANCOVA) for each post‐test score as a dependent variable with pre‐test scores as covariates, including a Bonferroni correction for multiple comparisons. Because the results were conceptually the same, we chose to report the MANOVA findings, with two exceptions described below.

Data screening indicated no missing data, and almost all variables were within tolerable ranges for skewness, with only the Long Term Memory pretest indicating a small positive skew. Finally, comparisons of pretest scores indicate groups were statistically equivalent on almost all measures (Associative Memory *t* = −.57, *p* = .57; Visual Processing *t* = −.21, *p* = .83; Auditory Processing *t* = .16, *p* = .87; Working Memory *t* = .66, *p* = .51; Long Term Memory *t* = −.35, *p* = .73; GIA *t* = .63, *p* = .54; Attention *t* = −.88, *p* = .39), with the exceptions of Logic and Reasoning (*t* = 2.33, *p* = .03) and Processing Speed (*t* = −2.04, *p* = .05), where the treatment group reported a lower mean score on the former (treatment = 100.70, control = 111.95) and a higher score on the latter (treatment = 87.35, control = 77.68). Because of the significant differences on these two measures, we provide results below from the aforementioned ANCOVA, in addition to the MANOVA, as the former presents the post‐test results after controlling for the pre‐tests.

## Results

As indicated in Table 2, participants in the treatment group consistently showed greater difference scores as compared to the control group on all measures. When examining the difference in difference scores, the greatest gap was evident between groups on Logic and Reasoning and GIA, with the smallest gaps present in Attention, Processing Speed, and Visual Processing. Moreover, subjects in the treatment group showed growth on all measures, whereas control group participants showed decreasing mean scores on four measures (Auditory Processing, Logic and Reasoning, Working Memory, and GIA). The greatest growth in the treatment group was evident in Long Term Memory, Associative Memory, and Logic and Reasoning, with the smallest growth in Attention and Visual Processing.

**Table 2:** Pre to post difference scores by group

	Control		Treatment		Difference
	Mean	*SD*	Mean	*SD*	M_T_ − M_C_
GIA	−5.11	8.93	21.00	13.49	26.11
Associative Memory	7.68	14.77	22.95	13.61	15.27
Visual Processing	4.26	10.30	10.85	9.75	6.59
Auditory Processing	−3.74	12.44	13.30	12.28	17.04
Logic and Reasoning	−7.21	10.87	21.10	18.50	28.31
Processing Speed	6.53	7.24	12.95	9.53	6.42
Working Memory	−7.68	19.66	13.05	15.11	20.73
Long Term Memory	6.95	13.05	28.20	22.38	21.25
Attention	3.17	7.34	5.06	8.12	1.89

**Figure 3:**
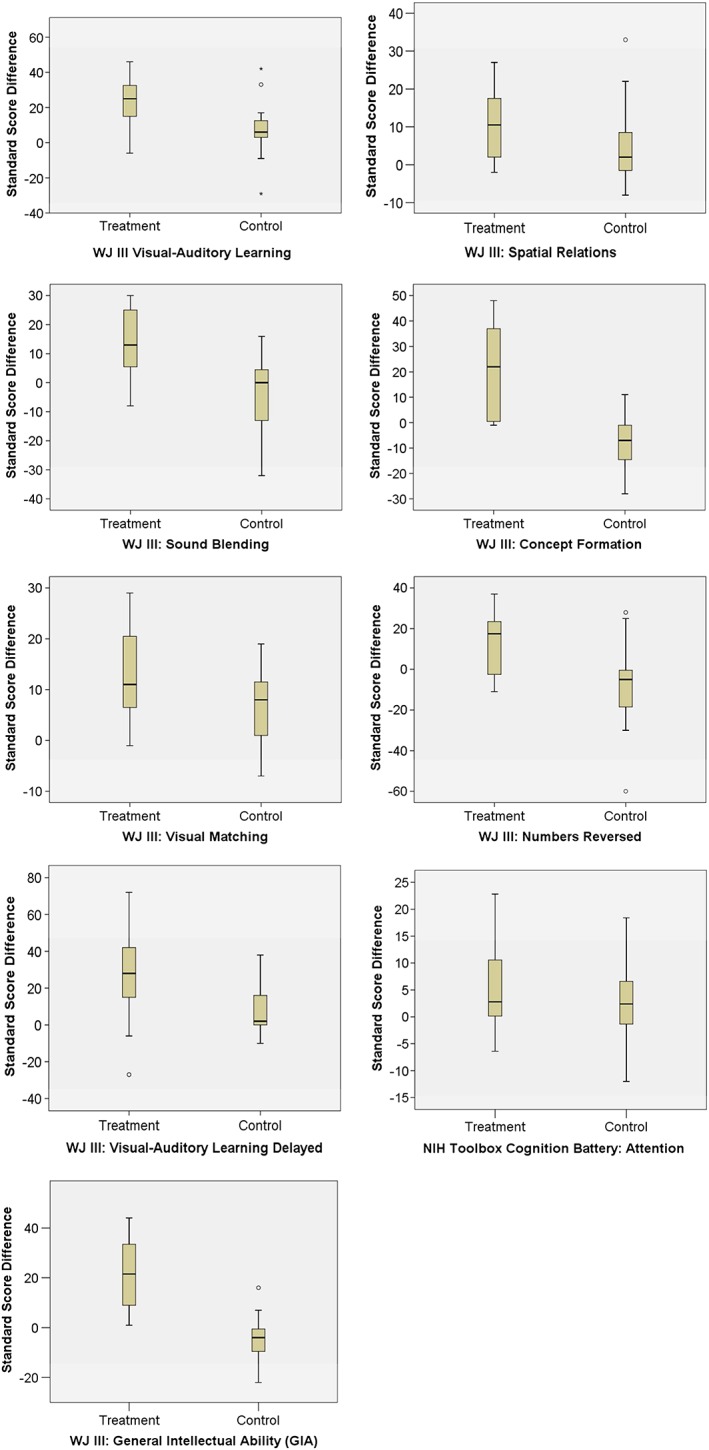
Distribution of change scores by group

Another way to visualize the differences is illustrated in Table 3. These data represent participants in each group whose scores were at or close to the mean difference score for each metric. As such, these can be thought of as average or representative participants for each group on each measure. These individual data demonstrate how much greater the growth was for treatment group participants as compared to those in the control. Among these representative participants, treatment students typically saw growth two to three times greater than that of those in the control. Notably, this is so despite treatment pre scores that almost always exceeded control pre scores. To illustrate treatment and control group differences in changes from pretest to post‐test, Figure 3 shows the distribution of change scores by group in the form of boxplots.

**Table 3:** Pre and post scores for a representative sample of selected participants at approximately the average of each measure's difference score

	Treatment			Control		
	Pre	Post	Diff	Pre	Post	Diff
Associative Memory	94	117	23	79	86	7
Visual Processing	96	107	11	93	97	4
Auditory Processing	116	133	17	105	104	−1
Logic and Reasoning	118	138	20	99	92	−7
Processing Speed	101	113	12	81	87	6
Working Memory	92	106	14	100	94	−6
Long Term Memory	90	117	27	78	86	8
GIA	126	146	20	109	103	−6
Attention	107	111	4	91	95	3

MANOVA results indicate an overall significant difference between treatment and control groups (*F* = 15.83, *p* = .00, partial η^2^ = .83), with pairwise comparisons indicating significant differences between groups on eight of nine measures. Table 4 illustrates the significance testing results for each assessment measure. The one difference that was not significant was in Attention. Turning to effect sizes indicating the magnitude of the significance, the greatest effect of the intervention was measured on GIA score, followed by Logic and Reasoning. Both measures saw extremely large effects. All three measures of memory also saw very large effects. The smallest effect was measured on Attention, then Visual Processing and Processing Speed, both of which saw medium to large effect sizes.

**Table 4:** Significance testing results for assessment measures

	*F*	*p*	*d*
GIA	50.20	0.00	2.92
Associative Memory	11.28	0.00	1.03
Visual Processing	4.21	0.05	0.64
Auditory Processing	18.53	0.00	1.37
Logic and Reasoning	33.49	0.00	2.60
Processing Speed	5.57	0.02	0.89
Working Memory	13.72	0.00	1.05
Long Term Memory	12.95	0.00	1.63
Attention	0.58	0.45	0.26

As for the ANCOVA analysis for Logic and Reasoning and Processing Speed, results indicate post‐test scores were significantly greater for treatment group subjects, after controlling for pre‐test scores. On Logic and Reasoning (*F* = 32.01, *p* = .000), treatment group students conditionally scored approximately 19 points greater than control participants (M_T_ = 123.08, *SE* = 2.35; M_C_ = 103.39, *SE* = 2.41). As for Processing Speed (*F* = 10.47, *p* = .003), treatment group students conditionally scored more than eight points greater than control participants (M_T_ = 96.67, *SE* = 1.81; M_C_ = 88.04, *SE* = 1.86).

**Figure 4:**
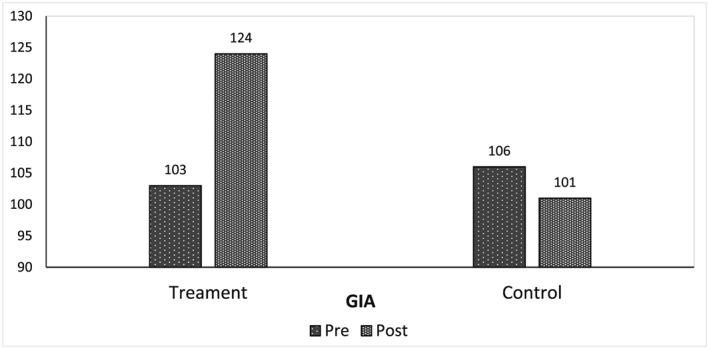
Comparison of treatment and control group mean pretest and posttest scores on General Intellectual Ability (GIA)

**Figure 5:**
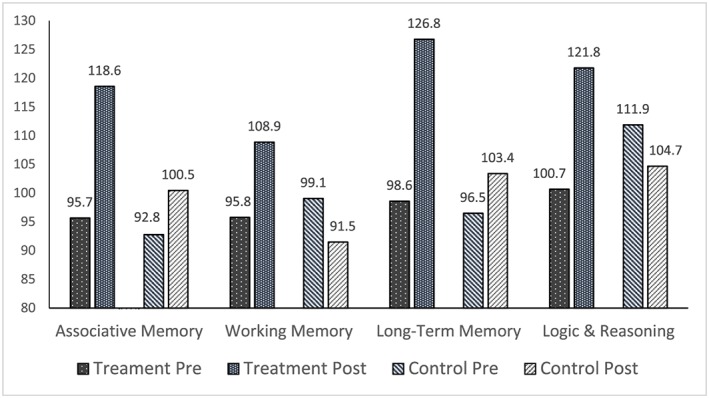
Comparison of treatment and control group mean pretest and posttest scores on memory and logic and reasoning

**Figure 6:**
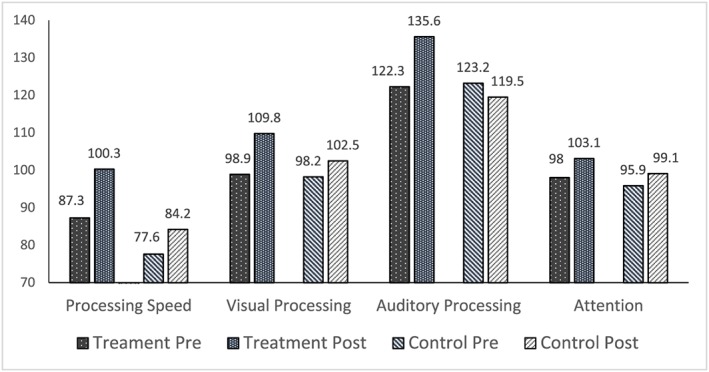
Comparison of treatment and control group mean pretest and posttest scores on processing speed, visual processing, auditory processing, and attention

In summary, the intervention produced statistically significantly greater growth on all measures except Attention. Those who received the intervention consistently showed growth from pretest to post‐test, while control group participants reported decreases on some measures. Finally, effect sizes were extremely large for two measures—Logic and Reasoning and GIA—and the large effect sizes for all three measures of memory were quite similar. Figures 4, 5, 6 show the between group pretest to post‐test differences.

## Discussion

In the current study, we tested the effects of a comprehensive cognitive training program delivered to children and adolescents in a one‐on‐one setting. Our research question asked if there is a statistically significant difference in GIA score, Associative Memory, Visual Processing, Auditory Processing, Logic and Reasoning, Processing Speed, Working Memory, Long Term Memory, and Attention between those who complete cognitive training and those who do not. The purpose for investigating the effects of this cognitive training program was to address two gaps in the cognitive training literature: the effects of a multidimensional, one‐on‐one cognitive training on multiple cognitive abilities and the effects of a comprehensive one‐on‐one cognitive training on GIA*.* Based on the comprehensive nature of the intervention, we predicted improvements in all nine measures for the treatment group.

The results of the study are consistent with the findings from an earlier quasi‐experimental study on the same program (Gibson et al., 2015) and add additional information to the literature with the inclusion of GIA and attention measures. Statistically significant differences between groups were found on all three measures of memory, on both auditory and visual processing, on processing speed, and on logic and reasoning. Further, the change in GIA score was significantly different between the two groups.

The positive effect of cognitive training on all three measures of memory generalized beyond the trained tasks because there are qualitative differences between the training and testing tasks. First, although Task 18 is an association task, it is also timed with a reverse sequence component. Second, unlike the WJ III associative memory test, Visual–Auditory Learning, there is no visual prompt provided in the associative memory training sessions after the initial associations between pictures and concepts have been learned. Further, associative memory training sessions are grounded in meaningful mnemonic device learning of real‐world associations rather than arbitrary images presented during the testing tasks. Third, the WJ III test of working memory, Numbers Reversed, is an auditory backwards span task. Alternatively, there are 12 training procedures that target working memory, none with a backwards span task. It is interesting to note that the backwards span task—which measures working memory capacity—is a powerful predictor of a student's ability to learn (Alloway & Copello, 2013). Thus, the generalization of the working memory training effects to working memory capacity is indeed a vital gain.

Four of the same training procedures that target working memory also target long‐term memory. The WJ III test for long‐term memory is a delayed administration of the associative memory test, Visual–Auditory Learning‐Delayed. With the exception of Task 18 described above, there are no training tasks that use associative memory tasks to target the development of long‐term memory. Thus, the gains in associative, working, and long‐term memory are more likely a function of generalized improvement in memory abilities rather than task‐specific performance improvements.

The differences between groups on the measure of processing speed also suggest generalized improvement. Twelve training tasks specifically target processing speed through the use of speeded tasks, tasks using a metronome, visual search and span tasks, computation tasks, memory‐building procedures, and tasks requiring sustained attention. One of the training tasks, Task 5, uses attention and visual discrimination to identify patterns in large blocks of numbers or letters. The more difficult levels of the task include sets of operations that the participant must perform on the items, such as circling one number, crossing out a different number, and placing a triangle around a third number. Although conceptually similar to the WJ III test of processing speed, Visual Matching, which requires the test‐taker to identify pairs of matching numbers on each line, the complexity of this training task engages multiple cognitive abilities and problem‐solving skills.

Visual and auditory processing differences were also significant between groups. The WJ III test to measure transfer of visual processing training, Spatial Relations, engages the participant in solving puzzles through mental rotation of pieces printed on the test. In the ThinkRx training program, there are nine procedures that target visual processing, three that target visual discrimination, five that target visual manipulation, and ten that target visualization. Unlike the testing task, none of the training procedures requires mental rotation of shapes. For example, in Task 19, participants use tangrams to recreate visual patterns from memory—a task that also targets visual memory, logic and reasoning, and attention. In Task 17, participants visualize a path and draw the route with their eyes closed. Given the qualitative differences between the testing and training tasks, gains in visual processing appear to be generalized improvements.

However, the difference between groups on auditory processing is probably best described as near transfer of the training effect. The WJ III testing task, Sound Blending, required participants to listen to individual sounds and specify the word the sounds make when blended together. In ThinkRx, there are six training tasks that target auditory processing. Because of the nature of the development of phonemic awareness, a primary way to learn sound blending is to practice blending sounds. Task 23 is the auditory processing training task that targets sound blending. Participants read the word, say the individual sounds, listen to a word, and say the individual sounds. Although the tasks seem similar, a key difference between the testing and training tasks is the use of nonsense words in training.

**Figure 7:**
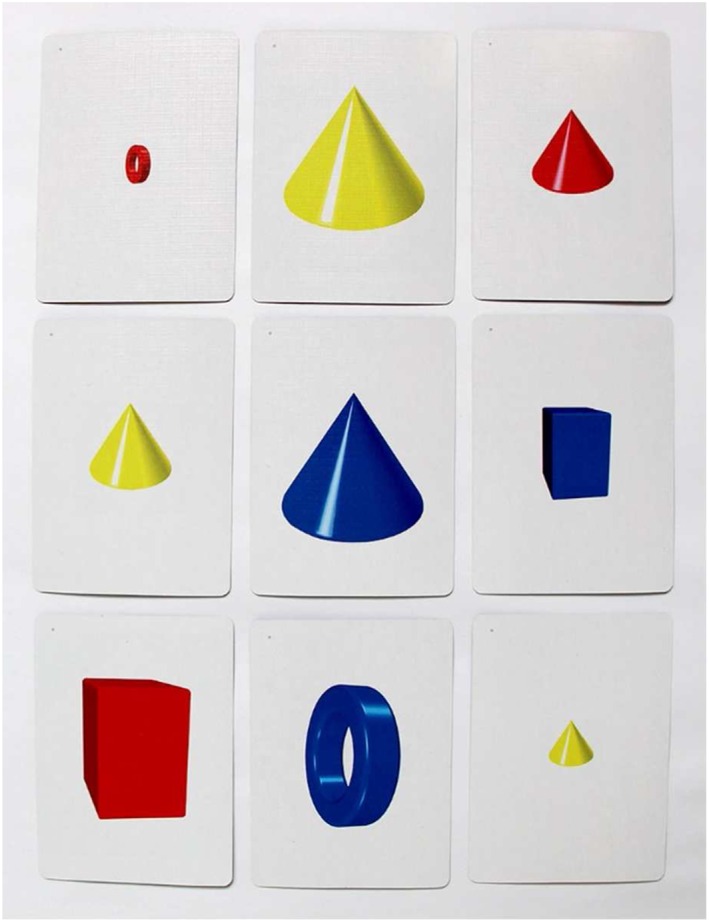
Example of a logic and reasoning training procedure. Participants must find a group of three cards where all of the card's features are the same or all different. In this example, the group includes the three cards with a vertical yellow cone, one small, one medium, and one large. All three cards share the same shape, color, and orientation, and all three cards are different sizes

The significant difference between groups on logic and reasoning may be a function of task generalization. The WJ III test for logic and reasoning, Concept Formation, is an inductive reasoning task asking participants to derive a rule for each item in a stimulus set. There are five training tasks that target logic and reasoning, including Tasks 14 and 15, which target deductive reasoning, congruence, part–whole relations, and diagramming. For example, Task 14 uses a deck of 81 cards containing small, medium, and large cones, rings, and boxes with three positional variations and three colors. Participants must create sets of three based on shared characteristics of the items. One level of this task is presented in Figure 7.

The difference in visual selective attention between groups as measured by the NIH Toolbox Flanker Test was not statistically significant. This may be because of the use of an unrelated arrows task during training of visual discrimination and selective attention that caused confusion for the treatment group during post‐testing. However, the results may instead be a reflection of the psychometric limitations of the measure. Although the NIH Toolbox Flanker Test received endorsement for use with ages 3–85 (Zelazo et al., 2013), the psychometric stability of the test for ages 8–15 is not firmly established. A validation of the Flanker Test with a pediatric population resulted in convergent validity of just .34 when compared with the D‐KEFS Inhibition test, and also found significant practice effects from repeated testing (Zelazo et al., 2013). Further, Akshoomoff et al. (2014) found significant ceiling effects in older children when conducting a large normative study on the cognition battery. Unfortunately, the use of the NIH Cognition Toolbox does not facilitate strong conclusions about the efficacy of the ThinkRx program on selective attention. However, it is important to note that Numbers Reversed subtest of the WJ III is a measure of broad attention. The difference between groups on the test of broad attention was indeed statistically significant.

There are applied implications to the findings from the current study. Cognitive training is applicable to both educational and clinical settings for remediating and strengthening cognitive abilities necessary for learning. Based on prior research that educational and personality characteristics of cognitive trainers do not significantly influence training outcomes (Moore, 2015), the LearningRx Corporation trained eight new people to serve as trainers for the current study. New trainers can learn the program in 25 instructional hours, and the curriculum and materials for each participant fit in a backpack. This simplicity in preparation for training and the portability of the materials suggests that the program can be delivered anywhere including clinics, schools, afterschool programs, tutoring centers, and homes. Given that 40% of high school seniors are not academically prepared for college (U.S. Department of Education, 2013) and that 2.4 million American children were identified as learning disabled in 2014 (Cortiella & Horowitz, 2014), one‐on‐one cognitive training may be a viable option for addressing the multiple cognitive deficits associated with learning problems.

The present study has some limitations. First, the results do not include longitudinal data on the lasting effects of cognitive training. However, this was the first phase of a larger year‐long study where researchers will collect follow‐up cognitive testing and academic achievement data. Next, some readers may be concerned that the use of a waitlist control group rather than an active control group may introduce the threat of expectancy effects. To mitigate the risk of expectancy effects, participants were not told that there was a waitlist control group. Instead, they were told that they were being assigned to either a summer or fall start for their training program. Further, prior research on expectancy effects in cognitive training studies has revealed that this is a minimal threat. Mahncke et al. (2006) tested the effect through the use of two control groups and concluded that the lack of difference between the two control groups suggests that there is no meaningful placebo effect with this type of study. Dunning et al. (2013) used a similar dual control group design and also concluded that experimental gains were not likely the result of expectancy effects. Burki, Ludwig, Chicherio, and Ribaupierre (2014) reported comparable results, finding no significant differences in training outcomes between active controls and no‐contact controls. Finally, two recent meta‐analyses of 35 cognitive training studies indicated no difference between types of control groups when compared to each other. One found significant treatment group gains regardless of the type of control group (Au et al., 2015), and the second also found that the type of control group did not have a significant influence on training effects (Peng & Miller, 2016).

A final limitation is that pretest group means on measures of logic and reasoning and processing speed were not homogenous. However, we opted not to drop the data from the logic and reasoning and processing speed tests because MANOVA is robust to the violation of homogeneity of covariance when group sizes are nearly equal (Tabachnick & Fidell, 2007; Warner, 2013), particularly when the significance value is not less than .001 (Field, 2009).

In addition to gathering longitudinal data and functional outcomes, future research should also incorporate neuroimaging data to assess how neural connections between brain regions are impacted by cognitive training. Research with a larger sample size and a like‐task comparison group might also be considered.

## Conclusion

In summary, the results of the current study provide additional support for the efficacy of the ThinkRx cognitive training program in improving cognitive skills in children ages 8–14. There were significant generalized improvements in seven cognitive skills—associative memory, working memory, long‐term memory, visual and auditory processing, logic and reasoning, and processing speed—as well as in the GIA cluster score. These findings also support the use of CHC Theory in the design of cognitive training programs to ensure multiple cognitive skills are targeted in the training tasks. There is much work to be done in the field of cognitive training research, and this study offers an important contribution to the knowledge base on cognitive training effects in children.
